# Detection assay of polymyxin resistance coding *mcr-1* gene based on CRISPR/Cas13a system

**DOI:** 10.3389/fcimb.2025.1553681

**Published:** 2025-06-05

**Authors:** Yingjie Song, Qiang Hu, Yao Han, Hongbo Liu, Zhenyang Huang, Mengwei Niu, Xue Dong, Kuocheng Yan, Li Jin, Hao Li, Yansong Sun

**Affiliations:** ^1^ State Key Laboratory of Pathogen and Biosecurity, Academy of Military Medical Sciences, Beijing, China; ^2^ Chinese PLA Center for Disease Control and Prevention, Beijing, China

**Keywords:** mcr-1, polymyxin, antibiotic resistance, CRISPR/Cas13a, lateral flow strip

## Abstract

**Introduction:**

Polymyxins are reserved as an ultimate defense against multidrug-resistant bacteria. The emergence of the polymyxin resistance gene *mcr-1* poses a potential risk for the treatment of severe infections caused by Gram-negative bacteria. Timely detection and monitoring the *mcr-1* gene are essential for guiding anti-infective therapy and controlling the spread of polymyxin resistance. Quantitative real-time PCR (qPCR) is one of the common methods for detecting resistance genes. However, qPCR has equipment dependency, and is not feasible in primary healthcare settings. Currently, there remains a lack of a highly sensitive and portable method for detecting the *mcr-1* gene.

**Methods:**

We established and optimized detection assays of the *mcr-1* gene based on CRISPR/Cas13a system and lateral flow strips. The detection method was preliminarily evaluated using clinical isolates from *Escherichia coli*, compared with qPCR.

**Results:**

The method for detecting the *mcr-1* gene based on the CRISPR/Cas13a system and lateral flow strips was established, with a detection limit of 100 copies/mL. This method demonstrated high analytical specificity, with no cross-reactivity detected in non-*mcr-1* and non-resistant strains. Among 36 clinical isolates, the method identified 31 strains as positive for the *mcr-1* gene, and had a 100% concordance rate with the results of qPCR.

**Conclusions:**

We established a detection method for the polymyxin resistance *mcr-1* gene based on the CRISPR/Cas13a system. This method enables visual readouts without instruments, making it potentially applicable to primary healthcare settings and field surveillance.

## 1 Introduction

The overuse and misuse of antibiotics have significantly contributed to the rise of bacterial resistance, particularly the emergence of multidrug-resistant bacteria, which poses great challenges for the treatment of clinical infectious diseases (Willyard, 2017; Nanayakkara et al., 2021; Thompson, 2022; Martin-Mateos et al., 2024). Previous research indicated that 4.71 million deaths were associated with bacterial resistance, including 1.14 million deaths attributable to antibiotic resistance (Collaborators, G.B.D.A.R, 2024). Polymyxin is a peptide antibiotic derived from *Paenibacillus polymyxa* that inhibits the growth and reproduction of most Gram-negative bacteria, such as *Escherichia coli* and *Pseudomonas aeruginosa* (Chiu et al., 2022). In clinical settings, the polymyxin formulations mainly include polymyxin B and polymyxin E. These agents exhibit equivalent bactericidal potency and spectrum. The main difference is that polymyxin B is administered directly as the active drug, whereas polymyxin E, also known as colistin, is administered in the prodrug form of colistimethate sodium (Nation et al., 2014; Kelesidis and Falagas, 2015). Due to its bactericidal mechanism, which directly disrupts the integrity of the bacterial outer membrane, polymyxin has excellent efficacy against multidrug-resistant bacteria and is recognized as a last line of defense against multidrug-resistant bacteria (Andersson et al., 2016; Murray et al., 2022). The polymyxin resistance gene *mcr-1* is generally located on bacterial plasmids and disseminates widely through horizontal gene transfer, enabling various bacterial strains to develop polymyxin resistance (Al-Tawfiq et al., 2017; Wise et al., 2018). Given the increasing public health threat posed by the emergence of the *mcr-1* gene, timely detection and surveillance of the *mcr-1* gene are critical for global antimicrobial resistance (AMR) control efforts.

Detection methods for the antibiotic-resistant phenotype of pathogenic bacteria include antimicrobial susceptibility testing (AST) and resistance genes detection. AST is the classic method for determining bacterial resistance phenotypes (Bartels et al., 2021; Tadesse et al., 2024), but it takes time to culture bacteria. It is a significant limitation for clinical decisions requiring rapid intervention. Quantitative real-time PCR (qPCR) is one of the common methods for detecting resistance genes, achieving rapid detection. Nevertheless, qPCR relies on specialized laboratories and sophisticated equipment, which limits its applicability in primary healthcare settings and hinders global AMR monitoring. Therefore, there is an urgent need for a sensitive and portable gene detection assay for antibiotic resistance.

The clustered regularly interspaced short palindromic repeats (CRISPR)/associated protein (Cas) system for nucleic acid detection has advanced rapidly (Gootenberg et al., 2017; Chen et al., 2018; Harrington et al., 2018; Kellner et al., 2019; Fozouni et al., 2021; Patchsung et al., 2023). The CRISPR/Cas13a system is characterized by targeted activation of additional cleavage activity. This system has been developed as a specific and high-sensitivity enzymatic reporter unlocking (SHERLOCK) assay, making it a prevalent choice for nucleic acid detection methods (Gootenberg et al., 2017; Kellner et al., 2019; Liu et al., 2017; Yin et al., 2022). In 2022, a study developed a highly sensitive CRISPR-based platform for *mcr-1* gene detection but relied on a real-time fluorescence detector, limiting its applicability in primary healthcare settings (Honda et al., 2011; Gong et al., 2022). By combining recombinase aided amplification (RAA) and lateral flow strips, CRISPR assay can achieve 1 copy/μL sensitivity within 60 minutes rivaling qPCR while eliminating equipment dependency (Li et al., 2021). Besides, RAA assay eliminates the need for expensive thermal cyclers through isothermal nucleic acid amplification, reducing equipment costs compared to PCR thermocycler. CRISPR-based lateral flow strips further minimize operational expenses by replacing fluorescence readers with visual readouts. Therefore, CRISPR-based lateral flow strips assay is a highly sensitivity, fast, low-cost method for the detection of nucleic acid.

In a previous study, we have developed a CRISPR-ERASE detection method that is simple, rapid, sensitive, and specific for the detection of SARS-CoV-2, with a detection limit of 1 copies/μL (Li et al., 2021). This method combines CRISPR/Cas13a system and lateral flow strips, and has been approved as a medical device product (20203400919) by the National Medical Products Administration. In this study, we have adapted this method to detect the *mcr-1* gene, which confers resistance to colistin. This novel application of CRISPR-ERASE enables rapid and cheap detection of the *mcr-1* gene, with potential for global surveillance of colistin resistance mediated by the *mcr-1* gene.

## 2 Materials and methods

### 2.1 Design and preparation of primers and crRNA

Sequences of the *mcr-1* gene (NG_050417.1) were downloaded from GenBank database. Conserved sequences of the *mcr-1* gene were identified by alignment and analysis using MEGA 7.0 software (Auckland, New Zealand). Four pairs of primers for isothermal amplification were then designed based on these conserved sequences. The T7 transcription promoter sequence was added at the 5’ end of the forward primer. Between the forward and reverse primers, we designed 3 crRNAs. The crRNA, probe, and primer sequences for the *mcr-1* gene were shown in **Supplementary Table 1**. The DNA sequence of the *mcr-1* gene was synthesized and cloned into pUC57 plasmid by Beijing Tianyihuiyuan Biotechnology Co., Ltd (Beijing, China), and the crRNA was prepared with reference to the literature (Li et al., 2020). The plasmid templates were absolutely quantified via digital PCR and then used as a template for analytical sensitivity and specificity detection experiments.

### 2.2 Samples preparation

In this study, 36 *Escherichia coli* strains were collected by the Chinese PLA Center for Disease Control and Prevention and shown in **Supplementary Table 2**. All strains were stored in 30% (w/v) glycerol broth at −80°C. The strains were incubated in Mueller-Hinton, followed by antimicrobial susceptibility test and plasmid extraction (TIANGEN, DP103). Plasmid templates were constructed by incorporating DNA fragment of the *mcr-1* gene. The recombinant plasmids were absolutely quantified and diluted 10-fold serially to yield 10^3^ copies/μL to 10^-1^ copies/μL.

### 2.3 Recombinase aided amplification

In accordance with the instructions provided with the DNA Isothermal Rapid Amplification Kit (Basic) (AMP Future Biotechnology Co., Ltd, WLB8201KIT), the amplification system consisted of 29.4 μL Buffer A, 2.5 μL Buffer B, 2 μL forward primer (10 nmol/L), 2 μL reverse primer (10 nmol/L), 9.1 μL non-enzymatic water, and 5 μL target DNA. The reaction system was incubated at a constant temperature of 39°C for 30 mins on a metal bath.

### 2.4 Fluorescence-based CRISPR assay

The crRNA mixed with the Cas13a enzyme to make the CRISPR/Cas13a-crRNA complex, which is used to incise the RAA product. The assay system consisted of 2 μL NTP Mix (2.5 mmol/L), 1 μL RNase Inhibitor (4 IU/μL), 0.5 μL T7 RNA polymerase (1 IU/μL), 0.5 μL HEPES (20 mmol/L), 0.25 μL MgCl_2_ (10 mmol/L), 10.75 μL enzyme-free water, 2.5 μL FAM-20U-BHQ1 (200 nmol/L), 1.5 μL crRNA (280 nmol/L), 1 μL Cas13a (45 nmol/L), and 5 μL RAA product. The fluorescence signals were collected every 2 min at 37°C to monitor the changes in fluorescence intensity. The threshold was determined by the mean fluorescence of the negative control group plus three times the standard deviation (Armbrecht et al., 2020).

### 2.5 CRISPR-ERASE assay

The total volume of the CRISPR-ERASE reaction system was 50 μL, and the mixture included 4 μL NTP Mix (2.5 mmol/L), 2 μL RNase Inhibitor (1.6 IU/μL), 1 μL T7 RNA polymerase (1 IU/μL), 1 μL HEPES (20 mmol/L), 0.5 μL MgCl_2_ (10 mmol/L), 26.5 μL DNase/RNase-Free Water, 5 μL FAM-20U-Biotin (2 nmol/L), 3 μL crRNA (2 nmol/L), 2 μL Cas13a (80 ng/L), and 5 μL RAA product. After incubation at 37°C for 30 min on a metal bath, the whole reaction system was transferred to ERASE lateral flow strip with a pipette gun. The reporter RNA molecule is labeled with FAM and biotin at its 5’ and 3’ ends, respectively. Colloidal gold particles coated with FAM antibodies are immobilized at the bottom of the test strip. When the target nucleic acid is present in the system, the activated Cas13a protein specifically cleaves the reporter RNA molecule. This cleavage releases the biotin moiety, which then binds to streptavidin encapsulated on the Test band. Simultaneously, the colloidal gold particles bind to the FAM moiety and the anti-FAM antibody encapsulated on the Control band, resulting in a visible signal only on the Control band. Conversely, when the target nucleic acid is absent, the reporter RNA molecule remains intact. In this case, the colloidal gold particles with FAM antibodies bind to streptavidin via the biotin molecule and also bind to the anti-FAM antibody, producing visible signals on both the Test and Control bands (Li et al., 2020). The results could be read with the naked eye after 5 mins.

### 2.6 Quantitative real-time PCR

A 10-fold gradient dilution of the *mcr-1* plasmid template was detected using a quantitative Real-time PCR probe kit purchased from Thermo Fisher. The qPCR primer and probe are used as reported in the literature and shown in **Supplementary Table 1** (Chabou et al., 2016). The reaction system was 10 μL TaqMan™ Fast Advanced Master Mix (2×), 0.5 μL forward primer (0.4 μmol/L), 0.5 μL reverse primer (0.4 μmol/L), 0.5 μL probe (0.4 μmol/L), 5.5 μL DNase/RNase-Free Water, and 3 μL target DNA. The qPCR conditions were as follows, the reaction mixtures were kept at 95°C for 30 s and subsequently put through 40 cycles of 95°C for 10 s and 60°C for 1 min, and the fluorescence signal was collected once at the end of each cycle. Real-time PCR was performed on serially diluted samples to measure cycle threshold (Ct) values. A standard curve was subsequently generated by plotting template concentrations (x-axis) against corresponding Ct values (y-axis). The limit of detection (LoD) was calculated as the minimum template concentration with Ct values statistically different from negative controls.

### 2.7 Antimicrobial susceptibility test

Polymyxin B susceptibility testing (Kangtai Biotechnology Co., Ltd, J1A079) was performed using the broth microdilution method, and results were interpreted according to European Committee on Antimicrobial Susceptibility Testing (EUCAST) breakpoints (EUCAST, 2025). Five colonies cultured for 18 h were taken by inoculation loop and put into sterile inoculum water, and the concentration was adjusted to 1.5*10^8^ cfu/mL. Take 10 μL of the conditioned bacterial solution and add it to the liquid medium of antimicrobial susceptibility testing and mix well, and then add 100 μL per well to each concave control in the antimicrobial susceptibility plate. Finally, the plates were incubated in an incubator at 37°C for 18 h and then removed for interpretation of the results.

### 2.8 Statistical analysis

The data were analyzed by normality tests and then unpaired two-tailed Student’s t tests using GraphPad Prism 8.0.2 software (GraphPad, Inc., La Jolla, CA, USA).

## 3 Results

### 3.1 Schematic of CRISPR-ERASE detection assay for *mcr-1* gene

**Figure 1:**
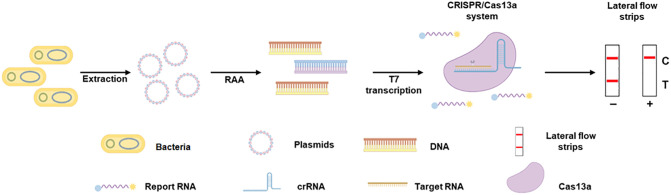
The schematic of CRISPR-ERASE assay for detecting the *mcr-1* gene. Plasmid DNA was extracted from *Escherichia coli*, amplified the *mcr-1* gene fragment by recombinase-assisted amplification (RAA) and transcribed into an RNA sequence. When CRISPR/Cas13a system recognizes the RNA target and degrades reporter RNA. The cleaved reporter RNA can be captured on a lateral flow strip and the results can be read with the naked eye using ERASE strips.

In this study, we developed a detection assay for the *mcr-1* resistance gene using CRISPR-ERASE technology. The schematic of the detection assay for the *mcr-1* gene is shown in **Figure 1**. Plasmid DNA was extracted from *Escherichia coli*, and the *mcr-1* gene fragment was amplified by recombinase-assisted amplification (RAA) and transcribed into an RNA sequence. When the CRISPR/Cas13a system recognizes the RNA target, it degrades the reporter RNA. The cleaved reporter RNA can be captured on a lateral flow strip and the results can be read with the naked eye using ERASE strips. A positive result is showed by the presence of only the Control band, while the appearance of both the Test and Control bands indicates a negative result.

### 3.2 Design and screening of RAA primers and crRNAs

**Figure 2:**
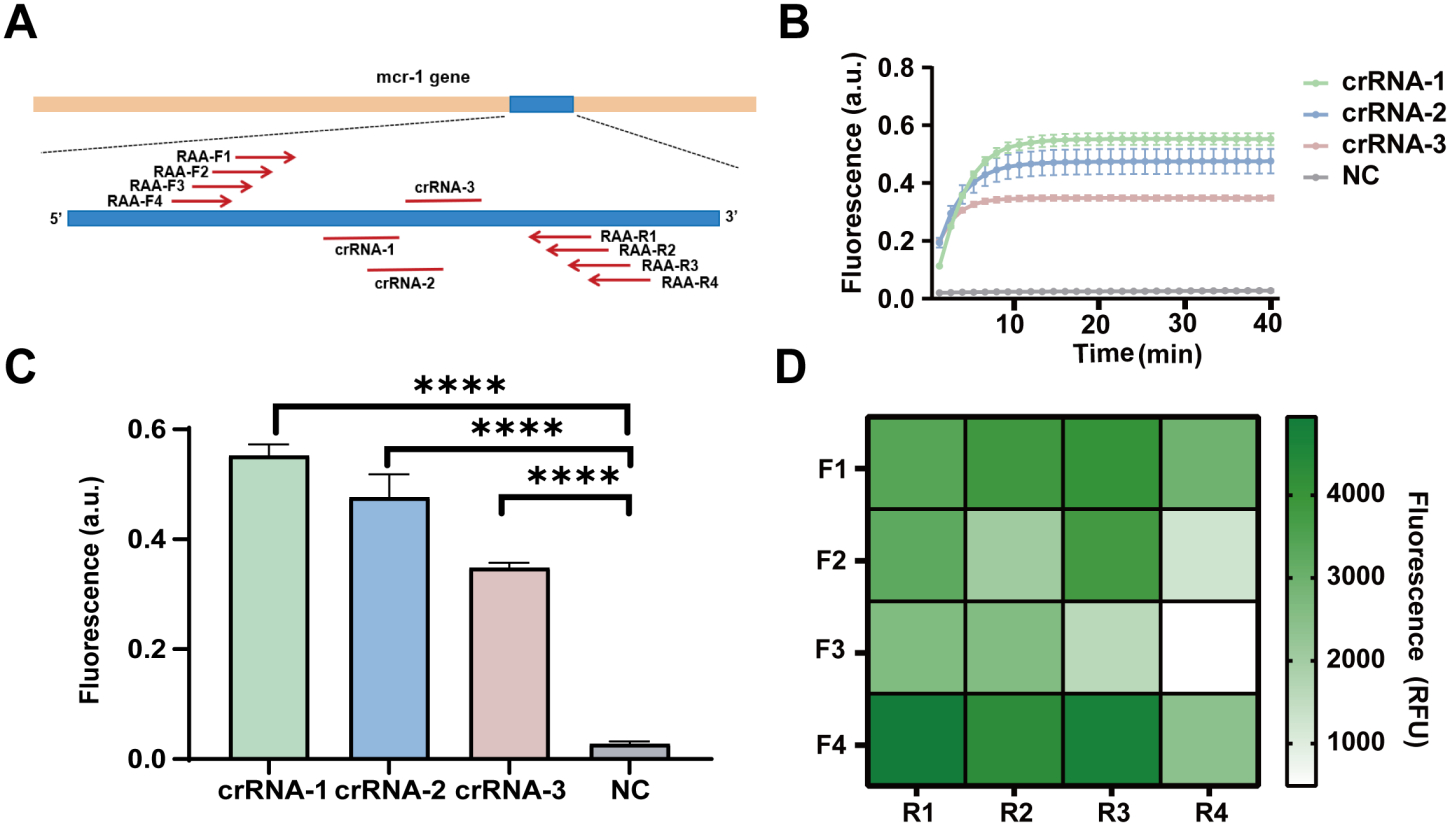
Design and screening of RAA primers and crRNAs for the detection of the *mcr-1* gene. **(A)** The schematic diagram of the design of RAA primers and crRNA. **(B)** The dynamic fluorescence curves within 40 min of 3 different crRNAs for CRISPR-ERASE assay (n = 3). **(C)** The fluorescence values of different crRNAs designed for the *mcr-1* gene were compared at the 40 min after detection reaction. ****p < 0.0001. The data were expressed as mean ± SEM (n = 3). **(D)** The fluorescence values produced at 40 min of CRSIPR fluorescence detection reaction with crRNA-1 and different RAA amplification primer combinations of *mcr-1* gene. The data were expressed as mean ± SEM (n = 3).

To screen efficient crRNA, we designed and screened 3 crRNAs and 4 pairs of RAA primers. The schematic representation of the positions of the crRNAs and RAA primers is shown in **Figure 2A**. Initially, we assessed the detection efficiency of the CRISPR/Cas13a system using three different crRNAs in conjunction with the same RAA product for the *mcr-1* gene. The results indicated that the fluorescence profile of the CRISPR/Cas13a system increases after the initiation of the reaction. At 60 minutes, the average fluorescence value for the crRNA-1 group showed a 19.64 ± 1.24-fold increase, while the crRNA-2 group exhibited a 17.02 ± 2.58-fold increase, and the crRNA-3 group demonstrated a 12.38 ± 0.54-fold increase, all compared to the negative control group. The crRNA-1 group displayed significantly higher fluorescence values compared to the other two groups (**Figures 2B, C**). Consequently, crRNA-1 was selected for further *mcr-1* gene detection.

To identify efficient RAA primers, we constructed 16 combinations by pairing four forward RAA primers with four reverse RAA primers targeting the *mcr-1* gene. These combinations were tested to simultaneously amplify plasmids containing the *mcr-1* gene fragment at a concentration of 10 copies/μL. The products were examined for fluorescence values using the CRISPR/Cas13a system with crRNA1. At 40 minutes, the primer combination F4R1 exhibited a fluorescence value of 4941 RFU, which was the highest among the 16 combinations (**Figure 2D**). Thus, the F4R1 primer was selected for subsequent *mcr-1* gene detection.

### 3.3 Limit of detection of the CRISPR-ERASE assay using the plasmid containing the *mcr-1* gene fragment

**Figure 3:**
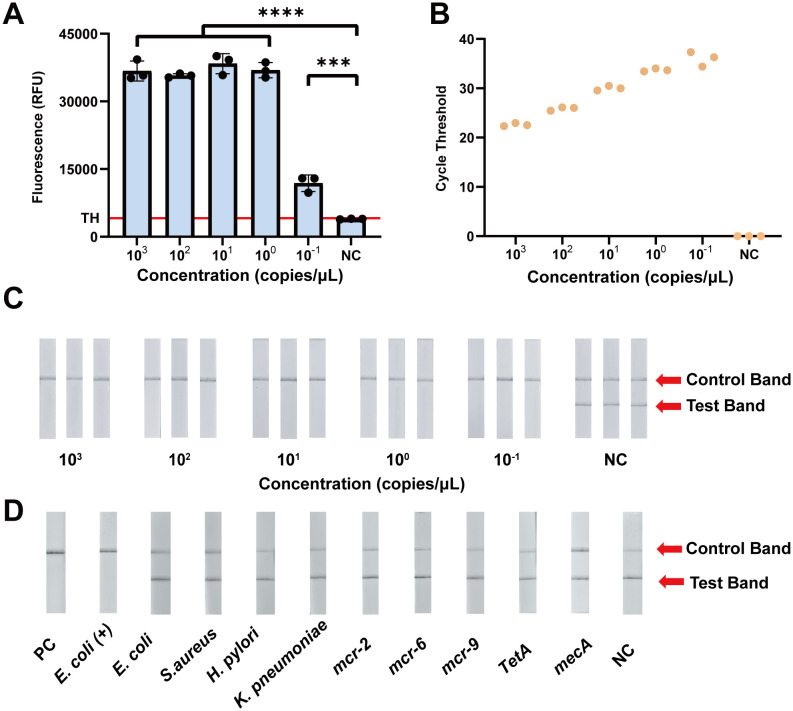
Analytical sensitivity and specificity evaluation of the CRISPR-ERASE assay for the detection of the *mcr-1* gene. **(A)** Comparison of fluorescence values produced by CRISPR fluorescence detection of *mcr-1* plasmid. TH means the threshold using the mean fluorescence value of the negative control group plus three times the standard deviation. ***p<0.001; ****p<0.0001. The data were expressed as mean ± SD (n = 3). **(B)** The results of qPCR at different *mcr-1* plasmid concentrations (10^3^ ~10^-1^ copies/μL) (n = 3). **(C)** The results of CRISPR-ERASE at different *mcr-1* plasmid concentrations (10^3^ ~10^-1^ copies/μL). Test band disappeared and control band was visible meaning that the test result is positive. Repeat each result three times. NC represents Negative Control. **(D)** CRISPR-ERASE assay was used to detect the *mcr-1* gene of different strains. PC represents Positive Control; *Escherichia coli (+)* represents *mcr-1*-positive *Escherichia coli*; *S.aureus* represents *Staphylococcus aureus*; *H. pylori* represents *Helicobacter pylori*; *K. pneumoniae* represents *Klebsiella pneumoniae* (n = 3).

With the most efficient primer pairs and crRNA, we evaluated the analytical sensitivity of the fluorescence-based CRISPR assay. Initially, we determined the Limit of Detection of the CRISPR-ERASE assay. As illustrated in **Figure 3A**, the fluorescence values of the group of 10^3^ to 10^-1^ copies/μL were significantly increased. To assess the experimental results quantitatively, we calculated the threshold using the mean fluorescence value of the negative control group plus three times the standard deviation. Fluorescence values above this threshold were classified as positive, while those below were classified as negative (Armbrecht et al., 2020; Ding et al., 2020; Sun et al., 2023). The fluorescence value of the CRISPR/Cas13a-based detection was calculated to be 4120.03 RFU. At a plasmid template concentration of 100 copies/mL, the fluorescence values for three replicate assays were 9765.63, 12903.47 and 12969.86 RFU, respectively, with all three replicates exceeding the threshold and thus classified as positive (**Figure 3B**). The LoD of the fluorescence-based CRISPR assay is 100 copies/mL.

To further assess the analytical sensitivity of CRISPR/Cas13a-based detection, we compared the LoD of the CRISPR-based assay and qPCR. At a plasmid concentration of 100 copies/mL, all three replicates had cycle threshold (CT) values of 37.30, 34.38, and 36.28, showing a positive results. qPCR detected the plasmid template containing the *mcr-1* gene at a concentration of 100 copies/mL (**Figure 3B**).

To avoid specialized instrumentation and achieve a portable detection method, we combined the CRISPR/Cas13a system with lateral flow strips. A plasmid containing the *mcr-1* gene fragment was subjected to a 10-fold gradient dilution (10^3^~10^-1^ copies/μL) and analyzed. At a template concentration of 100 copies/mL, only the Control band was visible on the ERASE strips across in all three replicate experiments, yielding consistently positive results (**Figure 3C**). These findings indicate that the LoD of the CRISPR-ERASE assay is 100 copies/mL.

### 3.4 Cross-reaction evaluation of the CRISPR-ERASE assay using the plasmid containing the *mcr-1* gene fragment

To evaluate the cross-reaction of CRISPR-ERASE, we analyzed the DNA of five pathogens: *mcr-1*-positive *Escherichia coli*, *Escherichia coli*, *Staphylococcus aureus*, *Helicobacter pylori*, and *Klebsiella pneumoniae*. pUC57 plasmids containing the *mcr-1* gene fragment were utilized as positive control templates, while pUC57 plasmids without the *mcr-1* gene fragment were utilized as negative control templates. As illustrated in **Figure 3D**, genomic DNA from the *mcr-1* carrying bacteria showed a disappearance of the Test band on the ERASE strips, while the Control band remained visible, indicating a positive result. In contrast, the ERASE strips for the remaining four bacterial pathogens displayed both Test and Control bands, confirming negative results. CRISPR-ERASE can specifically detect *mcr-1* producing bacteria without cross-reacting with the genomic DNA of the other four pathogens.

To evaluate the performance of CRISPR-ERASE with non-*mcr-1* genes, we compared the previously screened crRNA and RAA primers against the sequences of *mcr-2* to *mcr-11*. Since *mcr-2* and *mcr-6* exhibit higher homology with the target sequences, while the other variants show no match (Rodríguez-Santiago et al., 2021), we selected *mcr-2* and *mcr-6* for analytical specificity experiments. Given the higher prevalence of *mcr-9*, it was also included in cross-reaction experiments. Additionally, we tested two other common resistance genes, *tetA* and *mecA*, which confer resistance to tetracycline and methicillin, respectively. As illustrated in **Figure 3D**, we assessed cross-reactivity with five non-*mcr-1* genes and found no interference, thereby confirming the high analytical specificity of the assay.

### 3.5 Preliminary performance of CRISPR-ERASE assay in clinical isolates

**Figure 4:**
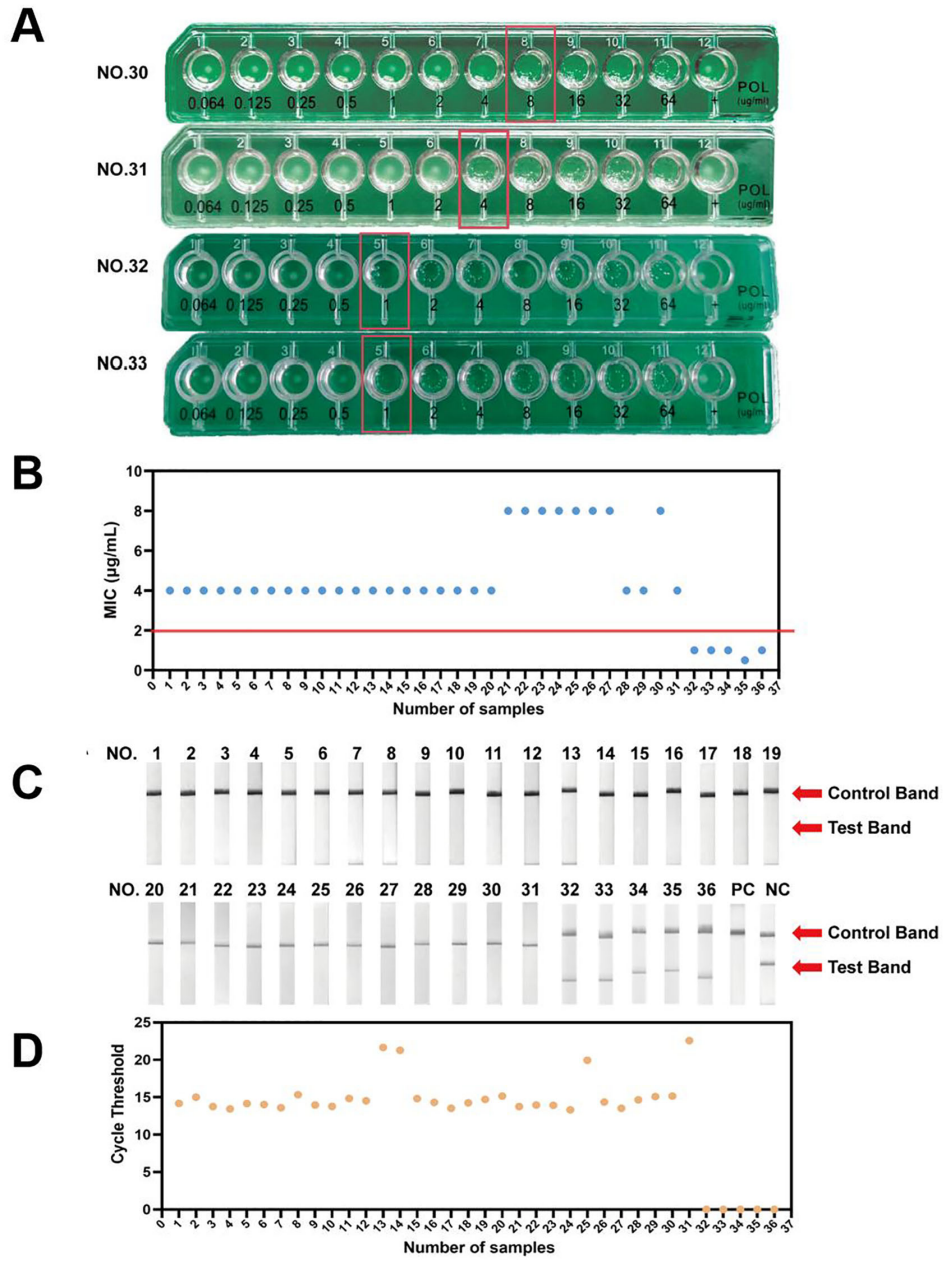
Preliminary validation of CRISPR-ERASE detection system to detect *mcr-1* gene in isolated *Escherichia coli* samples. **(A)** The results of antimicrobial susceptibility testing for NO.31-33 isolated samples. **(B)** The MIC results of antimicrobial susceptibility testing for 36 isolated samples. **(C)** The results of CRISPR-ERASE detection assay for 36 isolated samples. PC represents Positive Control.NC represents Negative Control. **(D)** The results of qPCR for 36 isolated samples.

For a preliminary evaluation of the CRISPR-ERASE assay, we tested 36 *Escherichia coli* strains isolated from clinical samples. We preformed antimicrobial susceptibility across a range of concentrations, determining the Minimum Inhibitory Concentration (MIC). For example, MIC values for clinical isolates No.30-33 were shown to be in the order of 4, 8, 1, and 1μg/mL (**Figure 4A**). According to EUCAST, an MIC of > 2 μg/mL is considered resistant, while an MIC of ≤ 2 μg/mL is classified as sensitive. As illustrated in **Figure 4B**, **Supplementary Data 1**, MIC values for clinical isolates No.1-19, 28, 29, and 31 were 4 μg/mL, while those for isolates No.20-27 and 30 were 8 μg/mL. Therefore, these 31 isolates were identified as polymyxin-resistant bacteria. In contrast, clinical isolates No.32, 33, 34, and 36 exhibited MIC values of 1 μg/mL, and isolate 35 had an MIC of 0.5 μg/mL, classifying these five isolates as polymyxin-sensitive bacteria.

Subsequently, we preliminarily evaluated the assay on 31 polymyxin-resistant bacteria samples and 5 polymyxin-sensitive bacteria samples. Clinical isolates numbered 1-31 showed only the Control band and no Test band on the strips, and all of these samples tested positive for the *mcr-1* gene. In contrast, clinical isolates numbered 32-36 displayed both Control band and Test bands on the strips and tested negative for the *mcr-1* gene (**Figure 4C**). The CRISPR-ERASE method identified that these 31 polymyxin-resistant samples were all samples carrying the *mcr-1* gene.

Furthermore, we performed qPCR on DNA samples extracted from 36 clinical isolates. Clinical isolates numbered 1-31 exhibited a maximum CT value of no more than 22.57, and all samples tested positive. In contrast, clinical isolates numbered 32-36 did not yield a readable CT value (**Figure 4D**). CRISPR-ERASE and qPCR results showed No.1-31 were tested positive for the *mcr-1* gene, No.32-36 were tested negative for the *mcr-1* gene. The results of quantitative real-time PCR were consistent with those of the CRISPR-ERASE method. Overall, the preliminary performance of CRISPR-ERASE assay in clinical isolates aligned with the findings from qPCR assay.

## 4 Discussion

Global efforts for the detection of antibiotic resistance genes have become increasingly urgent (Jiang et al., 2017; Wang et al., 2020; Kang et al., 2021; Zhang et al., 2022). In this study, we developed a rapid detection method for the *mcr-1* gene based on the CRISPR-ERASE assay. This assay detects plasmid templates containing the *mcr-1* gene at concentrations as low as 100 copies/mL, which exhibits a comparable LoD to qPCR using previously reported primers, suggesting a highly analytical sensitivity and accurate detection method for the *mcr-1* gene. Lateral flow strips offer a portable and visual detection method that is not constrained by external experimental conditions, making it particularly suitable for decentralized settings such as rural clinics or field surveillance programs. The assay specifically targets the *mcr-1* gene, with no cross-reactivity observed against non-resistant strains or other mcr variants, demonstrating high analytical specificity.

In our study, there are some limitations. First, while the CRISPR-ERASE assay avoids reliance on complex instruments, current nucleic acid extraction protocols still require centrifugation, which may hinder implementation in resource-limited regions. Future integration with direct lysis buffers could further streamline the workflow and save time. Second, validation was limited to *Escherichia coli* clinical isolates. Given the global prevalence of *mcr-1* in other Gram-negative pathogens such as *Klebsiella pneumoniae* and *Salmonella*, extending testing to these species is critical to confirm broad applicability. Third, although the assay’s multiplex potential was preliminarily explored, simultaneous detection of multiple resistance genes will require rigorous optimization to prevent crRNA cross-reactivity and ensure signal fidelity. Forth, the sample size of 36 clinical isolates is relatively small, which may potentially limit the robustness of diagnostic sensitivity and specificity. Further validation in a larger cohort and diverse clinical settings is needed to confirm its clinical utility.

Polymyxin resistance mechanisms extend beyond plasmid-borne *mcr-1* to include chromosomal mutations, with distinct pathways observed across bacterial species. While *Escherichia coli* predominantly acquires resistance through *mcr-1*-mediated plasmid transfer, *Klebsiella pneumoniae* primarily develops resistance via chromosomal mutations such as inactivation of the *mgrB* gene, a critical negative regulator of lipid A modification systems (Poirel et al., 2015). These species-specific resistance mechanisms highlight the complexity of polymyxin resistance in Gram-negative pathogens. From a clinical perspective, the CRISPR-ERASE assay has a potential impact on antimicrobial stewardship. The assay might be used to detect the inactivation of *mgrB* gene. Compared with traditional amplification and sequencing detection, this technology might detect inactivated genes more rapidly and at a lower cost. It is particularly relevant given that chromosomal-mediated resistance mechanisms like *mgrB* mutations are increasingly reported in clinical isolates worldwide (Poirel et al., 2015; Conceição-Neto et al., 2022). In veterinary or agricultural settings, this tool might support large-scale surveillance of *mcr-1* transmission at lower costs and shorter turnaround times. However, widespread adoption would require validation in diverse matrices and integration into existing diagnostic pathways. For instance, a positive CRISPR-ERASE result could trigger reflex testing by sequencing, while negative results might expedite de-escalation of reserve antibiotics. With its high-resolution genotyping capacity, CRISPR-ERASE can track the spread of resistance genes across different bacterial strains and healthcare settings. This helps in understanding the transmission dynamics of resistance and supporting for antimicrobial resistance epidemiology studies.

In the future, the development of the assay may focus on expanding target pathogens to include multidrug-resistant Gram-negative bacteria, developing multiplexed CRISPR panels for co-detection of high-priority resistance genes, and conducting field trials in low-resource hospitals to evaluate real-world feasibility. This technology not only addresses an urgent diagnostic gap but also aligns with global priorities to combat antimicrobial resistance through precision surveillance and targeted therapy.

In summary, we established a CRISPR-ERASE assay for rapid detection of the *mcr-1* resistance gene, achieving highly analytical sensitivity comparable to qPCR while eliminating reliance on specialized equipment. This platform enables timely identification of *mcr-1*-mediated colistin resistance, offering a potential tool to guide antimicrobial stewardship in clinical settings and support global surveillance efforts to curb the dissemination of plasmid-borne resistance.

## Data Availability

The original contributions presented in the study are included in the article/**Supplementary Material**. Further inquiries can be directed to the corresponding authors.
