# Supplementary material for: Detection assay of polymyxin resistance coding mcr-1 gene based on CRISPR/Cas13a system
Source: Front Cell Infect Microbiol. 2025 Jun 5;15:1553681. doi: 10.3389/fcimb.2025.1553681 (PMC12176852; doi:10.3389/fcimb.2025.1553681)
Supplement: Supplementary file 1 [file Table1.docx]

Supplementary Material

# Supplementary Data

**The results of antimicrobial susceptibility testing and qPCR on 36 clinical isolates.** NO means Number; MIC means Minimum Inhibitory Concentration.

NO.1 MIC (4 μg/mL)


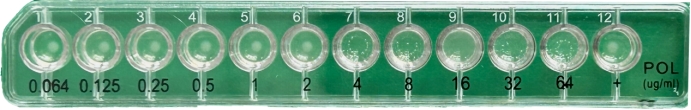


NO.2 MIC (4 μg/mL)


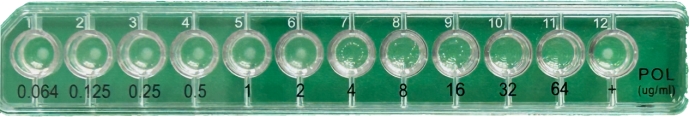


NO.3 MIC (4 μg/mL)


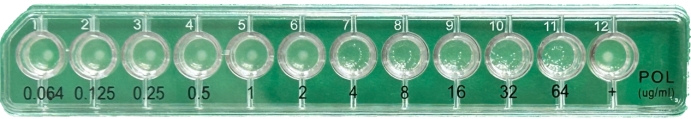


NO.4 MIC (4 μg/mL)


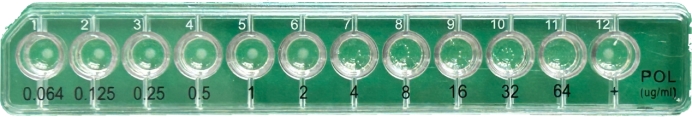


NO.5 MIC (4 μg/mL)


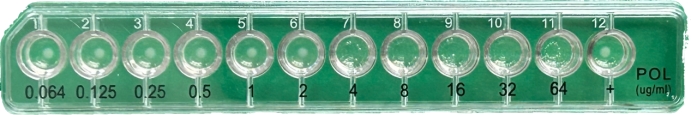


NO.6 MIC (4 μg/mL)


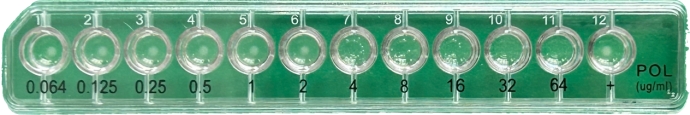


NO.7 MIC (4 μg/mL)


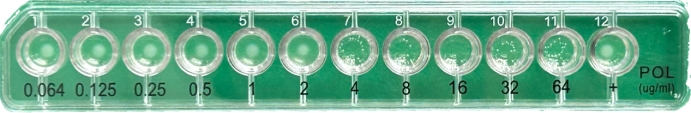


NO.8 MIC (4 μg/mL)


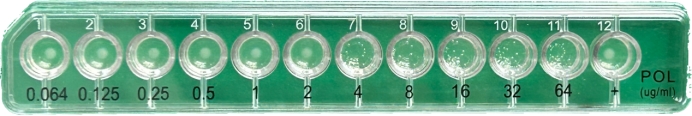


NO.9 MIC (4 μg/mL)


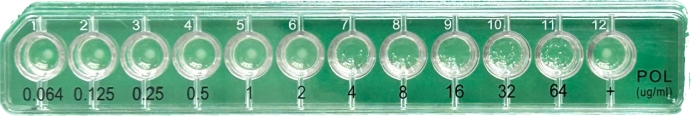


NO.10 MIC (4 μg/mL)


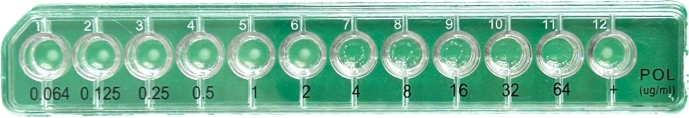


NO.11 MIC (4 μg/mL)


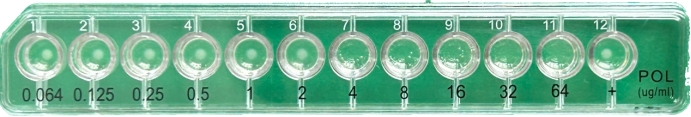


NO.12 MIC (4 μg/mL)


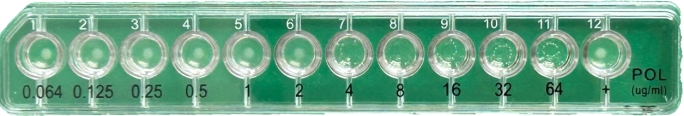


NO.13 MIC (4 μg/mL)


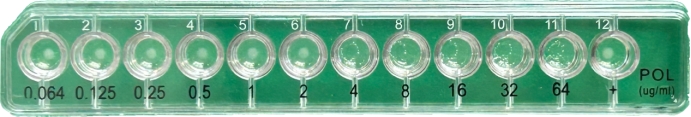


NO.14 MIC (4 μg/mL)


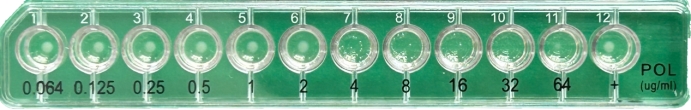


NO.15 MIC (4 μg/mL)


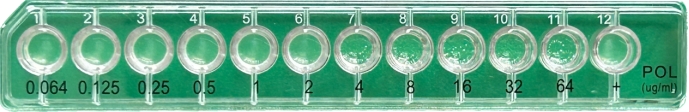


NO.16 MIC (4 μg/mL)


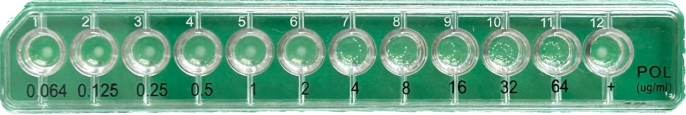


NO.17 MIC (4 μg/mL)


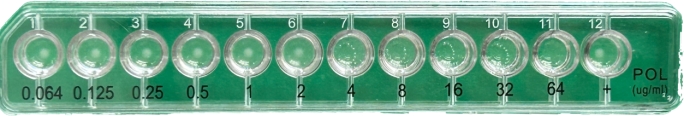


NO.18 MIC (4 μg/mL)


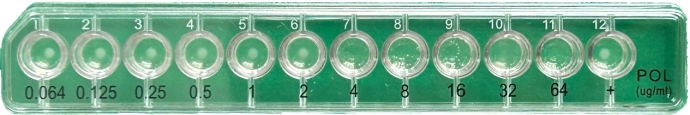


NO.19 MIC (4 μg/mL)


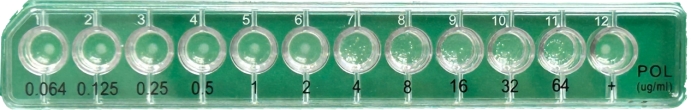


NO.20 MIC (8 μg/mL)


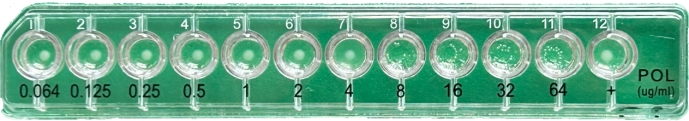


NO.21 MIC (8 μg/mL)


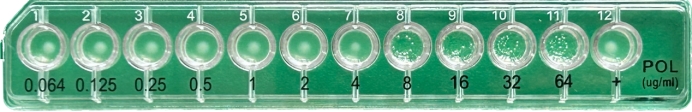


NO.22 MIC (8 μg/mL)


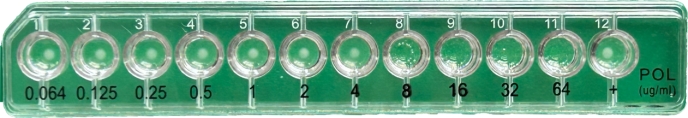


NO.23 MIC (8 μg/mL)


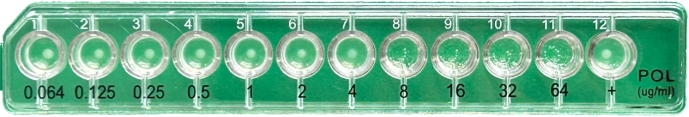


NO.24 MIC (8 μg/mL)


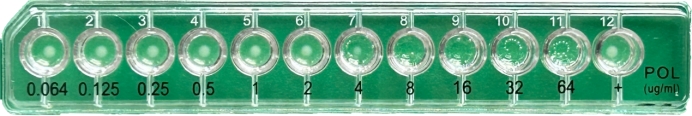


NO.25 MIC (8 μg/mL)


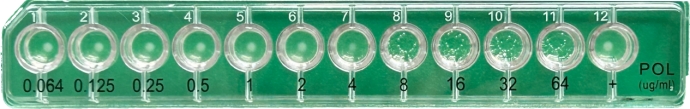


NO.26 MIC (8 μg/mL)


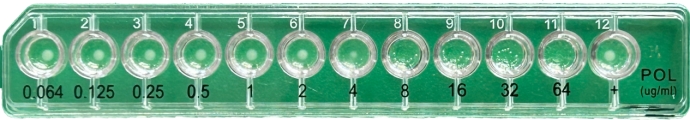


NO.27 MIC (8 μg/mL)


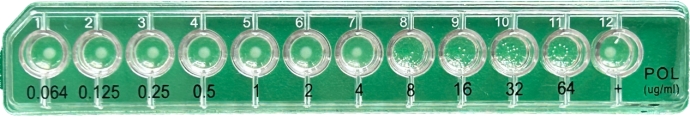


NO.28 MIC (4 μg/mL)


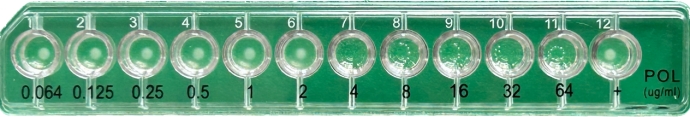


NO.29 MIC (4 μg/mL)


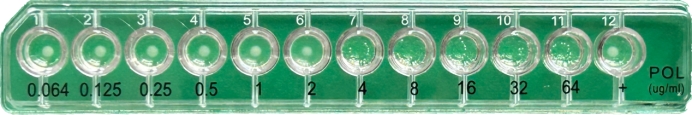


NO.30 MIC (8 μg/mL)


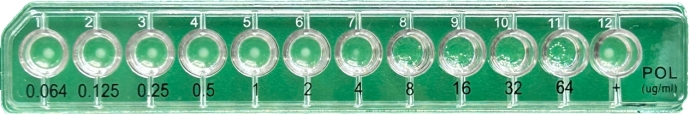


NO.31 MIC (4 μg/mL)


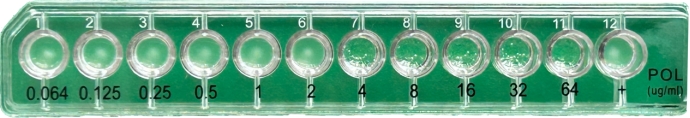


NO.32 MIC (1 μg/mL)


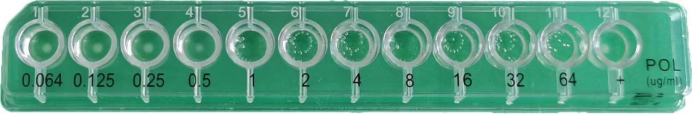


NO.33 MIC (1 μg/mL)


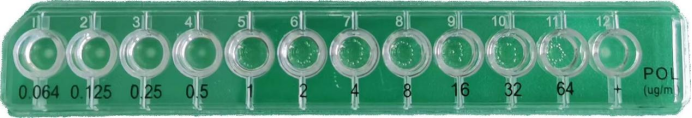


NO.34 MIC (1 μg/mL)


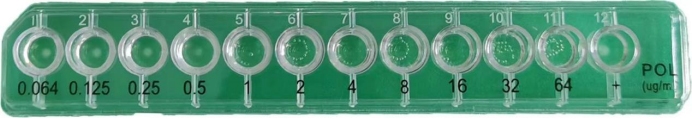


NO.35 MIC (0.5 μg/mL)


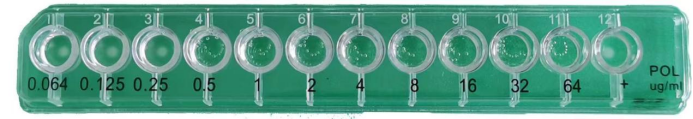


NO.36 MIC (1 μg/mL)


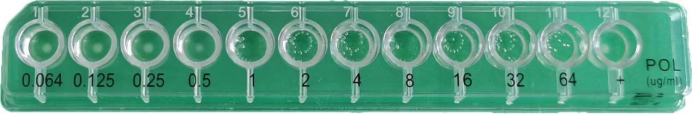


# Supplementary Figures and Tables

## Supplementary Tables

**Supplementary Table 1 The crRNA, probe, and primers sequence for *mcr-1* gene**

| Objects | Sequences (5′–3′) |
| --- | --- |
| FAM-20U-BHQ1 | FAM-UUUUUUUUUUUUUUUUUUUU-BHQ1 |
| FAM-20U-Biotin | FAM-UUUUUUUUUUUUUUUUUUUU-Biotin |
| RAA-Primer-F1 | AATTCTAATACGACTCACTATAGGGGTCGGTATGCTCGTTGGCTTAGATGACTTT |
| RAA-Primer-F2 | AATTCTAATACGACTCACTATAGGGGATGTCGGTATGCTCGTTGGCTTAGATGAC |
| RAA-Primer-F3 | AATTCTAATACGACTCACTATAGGGTGCCGCGATGTCGGTATGCTCGTTGGCTTA |
| RAA-Primer-F4 | AATTCTAATACGACTCACTATAGGGGAATGCCGCGATGTCGGTATGCTCGTTGGC |
| RAA-Primer-R1 | ACTTTTCATCATATCGCTTAAAATACGCAG |
| RAA-Primer-R2 | CAAACTTTTCATCATATCGCTTAAAATACG |
| RAA-Primer-R3 | TGGCAAACTTTTCATCATATCGCTTAAAAT |
| RAA-Primer-R4 | AATTTGGCAAACTTTTCATCATATCGCTTA |
| crRNA-1 | GGGATTTAGACTACCCCAAAAACGAAGGGGACTAAAACGCATATCTTTGCCGTTATTGGCAGCGAC |
| crRNA-2 | GGGATTTAGACTACCCCAAAAACGAAGGGGACTAAAACGGTGCAGCATGATCAGCATATCTTTGCC |
| crRNA-3 | GGGATTTAGACTACCCCAAAAACGAAGGGGACTAAAACTGATTGCCCATTTGGTGCAGCATGATCA |
| qPCR-F | GGGTGTGCTACCAAGTTTGCTT |
| qPCR-R | TATGCACGCGAAAGAAACTGGC |
| qPCR-Probe | 6-FAM-GCGCTGATTTTACTGCCTGTGGTG-TAMRA |

**Supplementary Table 2 The information of 39 strains.**

| NO | SCDC Number | Species | region | institution | Sampling time | Specimen |
| --- | --- | --- | --- | --- | --- | --- |
| 1 | SH12E009 | *E. coli* | Shanghai | hospital | 2012/08/13 | Faeces |
| 2 | SH12E308 | *E. coli* | Shanghai | hospital | 2012/11/21 | Faeces |
| 3 | SH13E058 | *E. coli* | Shanghai | hospital | 2013/05/26 | Faeces |
| 4 | SH13E099 | *E. coli* | Shanghai | hospital | 2013/07/01 | Faeces |
| 5 | SH13E201 | *E. coli* | Shanghai | hospital | 2013/08/12 | Faeces |
| 6 | SH13E393 | *E. coli* | Shanghai | hospital | 2013/10/10 | Faeces |
| 7 | SH13E397 | *E. coli* | Shanghai | hospital | 2013/10/10 | Faeces |
| 8 | SH13E519 | *E. coli* | Shanghai | hospital | 2013/09/12 | Faeces |
| 9 | SH13E569 | *E. coli* | Shanghai | hospital | 2013/10/15 | Faeces |
| 10 | SH13E570 | *E. coli* | Shanghai | hospital | 2013/10/21 | Faeces |
| 11 | SH14E003 | *E. coli* | Shanghai | hospital | 2014/01/05 | Faeces |
| 12 | SH14E004 | *E. coli* | Shanghai | hospital | 2014/01/09 | Faeces |
| 13 | SH14E023 | *E. coli* | Shanghai | hospital | 2014/03/31 | Faeces |
| 14 | SH14E027 | *E. coli* | Shanghai | hospital | 2014/04/08 | Faeces |
| 15 | SH14E032 | *E. coli* | Shanghai | hospital | 2014/04/13 | Faeces |
| 16 | SH14E039 | *E. coli* | Shanghai | hospital | 2014/04/21 | Faeces |
| 17 | SH14E066 | *E. coli* | Shanghai | hospital | 2014/05/18 | Faeces |
| 18 | SH14E080 | *E. coli* | Shanghai | hospital | 2014/05/27 | Faeces |
| 19 | SH14E227 | *E. coli* | Shanghai | hospital | 2014/07/14 | Faeces |
| 20 | SH14E268 | *E. coli* | Shanghai | hospital | 2014/07/23 | Faeces |
| 21 | SH14E280 | *E. coli* | Shanghai | hospital | 2014/07/28 | Faeces |
| 22 | SH14E587 | *E. coli* | Shanghai | hospital | 2014/10/17 | Faeces |
| 23 | SH14E620 | *E. coli* | Shanghai | hospital | 2014/11/05 | Faeces |
| 24 | SH14E621 | *E. coli* | Shanghai | hospital | 2014/11/05 | Faeces |
| 25 | SH14E634 | *E. coli* | Shanghai | hospital | 2014/11/15 | Faeces |
| 26 | SH14E666 | *E. coli* | Shanghai | hospital | 2014/12/08 | Faeces |
| 27 | SH14E763 | *E. coli* | Shanghai | hospital | 2014/10/13 | Faeces |
| 28 | SH15E025 | *E. coli* | Shanghai | hospital | 2015/03/02 | Faeces |
| 29 | SH15E070 | *E. coli* | Shanghai | hospital | 2015/04/25 | Faeces |
| 30 | SH15E075 | *E. coli* | Shanghai | hospital | 2015/05/04 | Faeces |
| 31 | SH15E694 | *E. coli* | Shanghai | hospital | 2015/01/06 | Faeces |
| 32 | EcoR01 | *E. coli* | Shanghai | hospital | / | Faeces |
| 33 | EcoR02 | *E. coli* | Shanghai | hospital | / | Faeces |
| 34 | EcoR03 | *E. coli* | Shanghai | hospital | / | Faeces |
| 35 | EcoR04 | *E. coli* | Shanghai | hospital | / | Faeces |
| 36 | EcoR05 | *E. coli* | Shanghai | hospital | / | Faeces |
| 37 | Jpj026 | *S. aureus* | Guangdong | market | 2008/04/07 | salad |
| 38 | / | HP | Shanghai | hospital | / | / |
| 39 | KpnR01 | KP | Shanghai | hospital | / | / |
